# Clinical outcomes of fibrin glue assisted hemostasis in persistent intraoperative optic disc bleeding

**DOI:** 10.1186/s40942-026-00801-w

**Published:** 2026-02-06

**Authors:** Saarang Hansraj, Thirumalesh MB, Brijesh Takkar, Vivek Pravin Dave, Ritesh Narula, Srishti Raksheeth Ramamurthy, Mudit Tyagi

**Affiliations:** 1 Smt. Kanuri Santhamma Centre for Vitreoretinal Diseases, Anant Bajaj Retina Institute, Saroja A Rao Uveitis Centre, Kallam Anji Reddy Campus, L V Prasad Eye Institute, Hyderabad, 500034 India; 2 Narayana Nethralaya, Bangalore, India; 3 Indian Health Outcomes, Public Health and Economics Research Centre (IHOPE), LV Prasad Eye Institute, Hyderabad, India

**Keywords:** Fibrin glue, Hemostasis, Optic disc bleed, Vitreous hemorrhage, Tractional retinal detachment, Diabetic retinopathy.

## Abstract

**Purpose:**

Persistent optic disc bleeding during pars plana vitrectomy (PPV) is difficult to manage. This study aims to explore the use of fibrin glue as a novel agent to achieve hemostasis in such eyes.

**Methods:**

Interventional case series of patients with vitreous hemorrhage (VH) or tractional retinal detachment (TRD) due to proliferative diabetic retinopathy, retinal vein occlusion or retinal vasculitis, in whom fibrin glue was used intraoperatively for hemostasis. All the patients were followed up for 1 month.

**Results:**

14 eyes of 14 patients were included, of which 12 (85.7%) were male with mean age of 52 years. Surgery was performed in 10 eyes (71.4%) for VH and 4 eyes (28.6%) for TRD. The mean best corrected visual acuity (BCVA) improved from 1.33±0.53 Logarithm of the Minimum Angle of Resolution (logMAR) (20/428), to 0.42± 0.35 logMAR (20/53) 1 month after surgery (*P* = 0.002), 0.43± 0.32 logMAR (20/54) 3 months after surgery (*p* = 0.003) and 0.36±0.34 logMAR (20/46) at a mean of 7 months (*p* = 0.002) after the surgery. Post operative vitreous cavity hemorrhage occurred in 1 eye (7.1%), which did not require a repeat surgery.

**Conclusions:**

This report demonstrates the efficacy of fibrin glue used during vitrectomy in controlling persistent optic disc hemorrhage in a cohort of diverse etiologies.

**Supplementary Information:**

The online version contains supplementary material available at 10.1186/s40942-026-00801-w.

## Abstract (highlights)

Persistent intraoperative optic disc bleeding is difficult to manage with adverse outcomes.

Fibrin glue is a short acting bioadhesive, which has shown favorable outcomes when used in rhegmatogenous retinal detachment and macular hole surgeries.

Our prospective interventional study shows that the use of fibrin glue is a valuable technique to manage optic disc bleeders, allowing for better and faster visual recovery, while reducing the incidences of rebleeding and reoperations.

Of the 14 eyes included in our study, post-operative vitreous cavity hemorrhage occurred in just one case, which resolved spontaneously, and did not need any reoperation.

**Supplementary Information:**

The online version contains supplementary material available at 10.1186/s40942-026-00801-w.

## Introduction

Achieving hemostasis during pars plana vitrectomy for vitreous haemorrhage (VH) or tractional retinal detachment (TRD) is challenging yet essential, allowing effective clearing of membranes and hemorrhage. Control of optic disc bleeders can be especially challenging since these are not amenable to endodiathermy [1]. One of the most common complication after PPV is persistent or recurrent VH after the primary surgery and has been termed as post-operative vitreous cavity hemorrhage (POVCH) and has been reported in 7–63% of patients. The various causes of POVCH include residual or recurrent vascular membranes, intra-operative iatrogenic break, insufficient endolaser photocoagulation, immediate post-operative hypotony, entry site neovascularization, and anterior hyaloid fibrovascular proliferation [2, 3].

POVCH can be present from the first post-operative day (persistent- 20–63% of patients), or can occur within the first 4–6 weeks (early-5%) or thereafter (delayed- 8%). Persistent and early vitreous cavity hemorrhage are mainly secondary to incomplete intraoperative hemostasis, bleeding from dissected fibrovascular tissue and release of erythrocytes from residual peripheral vitreous gel and iatrogenic injury to the retina or retina vessels. Immediate postoperative hypotony also increases the risk of immediate post-operative vitreous hemorrhage as does pre-operative neovascularization of the iris. Previous lower extremity amputation and failure to take prescribed antihypertensives are also associated with increased risk of persistent or early POVCH. Delayed POVCH (occurring 3 months or greater after surgery) occurs in approximately 8% of patients. There are 3 common causes for delayed POVCH: firstly, from residual fibrovascular membranes; secondly, from sclerotomy entry site fibrovascular ingrowth, and thirdly, from reproliferative retinal or ciliary body neovascularisation [3].

Adequate removal of vascular membranes overlying the disc is difficult and often poses a risk of persistent disc bleed which can lead to intraoperative as well as post-operative bleeding. It has generally been seen associated with early POVCH [2]. In this prospective interventional case series we describe the outcomes of a novel surgical technique of fibrin glue assisted hemostasis for the management of uncontrolled intra-operative disc bleed.

## Methods

This was a prospective interventional study of all patients who underwent PPV for VH or tractional retinal detachment (TRD) and required the use of fibrin glue for persistent intraoperative optic disc hemorrhage between January 2023 and January 2024. The study was approved by the institute review board and adhered to tenets of declaration of Helsinki. A written informed consent was taken from each patient.

The main outcomes were the incidence of persistent or early POVCH (POVCH on post operative day 1 or any POVCH occurring within 1 month of the primary surgery respectively) and improvement in mean best corrected visual acuity (BCVA). Secondary outcome measures were complications like incidence of epiretinal membranes (ERM) and any post-operative inflammation. All outcomes were assessed till 1 month after surgery.

All patients were examined prior to the surgery and on post-operative day 1, day 7 and day 30. At each visit BCVA was assessed with Snellen visual acuity charts, along with a comprehensive examination. Any patient with duration of follow up less than 1 month was excluded from the study.

Patient care was coordinated with internists throughout for good control of the underlying etiologies of proliferative retinal vasculopathies.

### Surgical technique

**Fig. 1:**
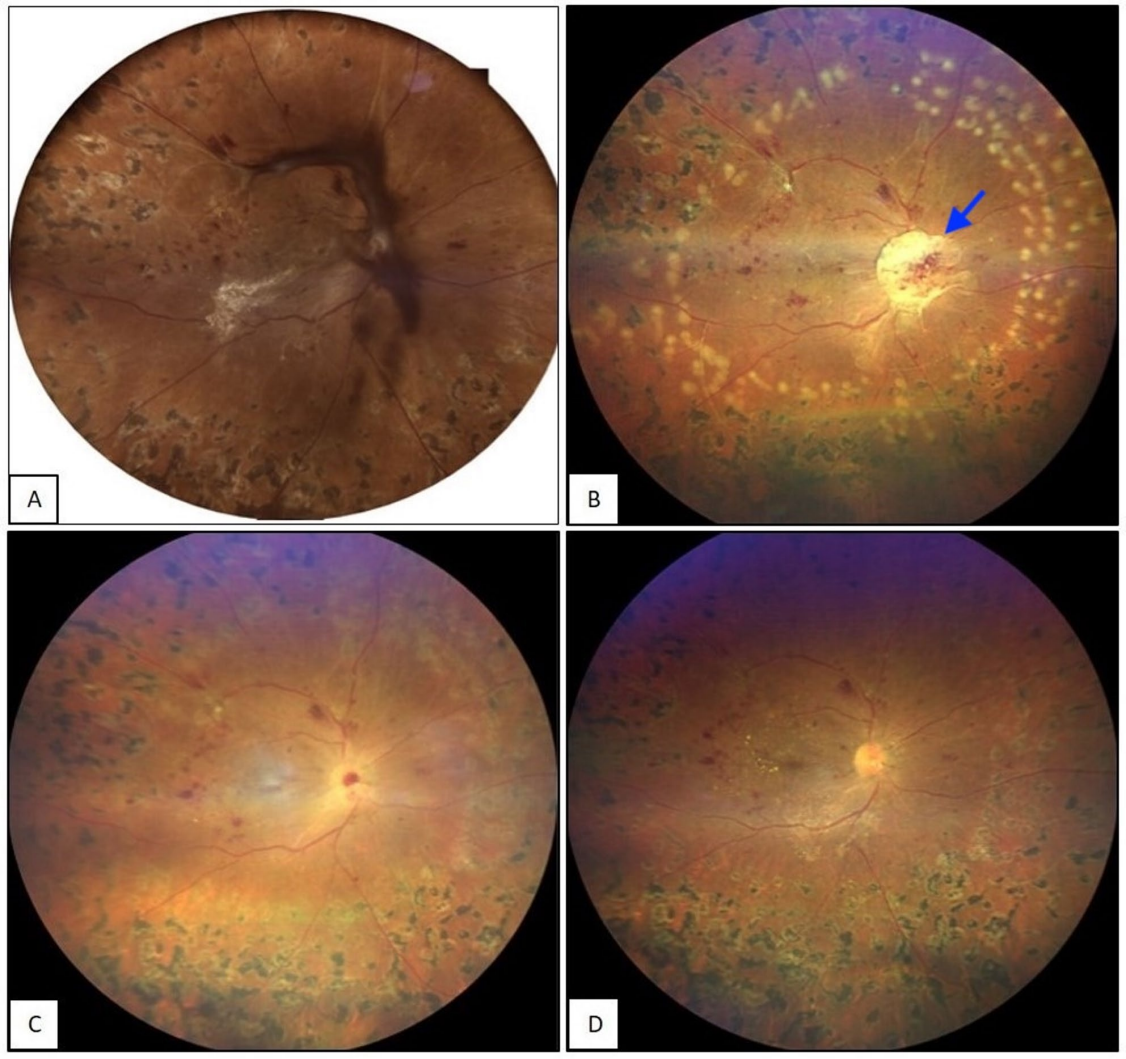
(**A**) Color fundus photograph of the right eyes shows tractional retinal detachment, with membrane over the disc and vitreous hemorrhage, requiring pars plana vitrectomy. (**B**) The widefield true color image taken on post-operative day 1 shows an attached retina, with a fibrin clot over the disc (arrow). The case was closed under balanced salt solution with no endotamponade. No persistent hemorrhage is noted. (**C**) The widefield true color image taken 1 week after the surgery shows that the fibrin clot is resolving. There has been no recurrent vitreous cavity hemorrhage. A small residual hemorrhage from the pre operative neovascularisation at the optic disc can be seen with no extension onto the retina, with a completely clear media. (**D**) Widefield true color image taken 1 month after the surgery shows that the fibrin clot has regressed, and retina is attached, with no active neovascularization

All patients underwent surgery under peribulbar anesthesia. Standard 25-gauge PPV was performed by trained vitreoretinal surgeons (BT, MT, RN, TMB and VPD). The vascular membranes overlying the disc were dissected and removed using the cutter or intraocular forceps. Following this manoeuvre, if disc bleeding was noted then attempts at hemostasis were attempted by elevating the infusion pressure to 60 mm Hg and subsequent clearing of the bleed with flute needle. However if the bleeding persisted even after multiple attempts of elevating intraocular pressure and fluid air exchange, fibrin glue was injected at the bleeding site(s). The technique of fibrin glue injection has been previously described for rhegmatogenous retinal detachment surgery [4, 5]. Fibrin glue for hemostasis in cases of disc bleeding was also earlier described by our group [6]. Under air, 1 drop of the thick component (thrombin) of glue was injected with a 1 cc tuberculin syringe. The thin component (fibrinogen) was injected in a controlled manner using a micro dose injector (MedOne Surgical Inc., Florida, USA) connected to viscous fluid injection (VFI) mode on the vitrectomy machine. At a pressure of 20 mm Hg using VFI, a single drop of thin component was subsequently injected. This technique prevents uncontrolled injection of large quantity of thin component which is likely to occur with the routine injection technique. After 3 min, a polymerized gel-clot was formed which was reposited over the disc with a flute needle to prevent further bleed. (Fig. 1) The cases were closed either under fluid or the tamponading agent of choice of the surgeon.

### Statistical analysis

Analysis was done using Microsoft Excel version 2021 and the GraphPad statistical calculator (https://www.graphpad.com). All visual acuity measurements were converted from Snellen chart values to the Logarithm of the Minimum Angle of Resolution (logMAR). A Wilcoxon Signed Rank test was used to calculate change in mean BCVA. A two tailed p value less than equal to 0.05 was considered statistically significant.

## Results

14 eyes of 14 patients were enrolled in the study.

### Demography

The mean age of patients was 52 years (SD +/-12 years) with 12 males (85.7%) and 2 females (14.2%).

### Baseline clinical features

Out of 14 eyes, 11 eyes (78.5%) had PDR, 1 eye (7.1%) had VH after a branch retinal vein occlusion (BRVO), 1 eye (7.1%) had tubercular vasculitis and 1 eye (7.1%) had VH and vasculitis after resolution of Acute Retinal Necrosis (ARN). 10 of the eyes (71.4%) required surgery for VH and 4 eyes (28.6%) for TRD. None of the patients had received any anti VEGF injection preoperatively.

Upon assessing systemic co morbidities it was seen that 2 patients (14.2%) had diabetes mellitus, 1 patient (7.1%) had hypertension, 9 patients (64.2%) had both hypertension and diabetes mellitus and 1 patient had pulmonary tuberculosis (7.1%). 2 (14.3%) of the patients had nutritional anaemia as well.

### Visual outcomes

The mean BCVA prior to surgery was 1.33 logMAR (SD +/- 0.53 logMAR, Snellen 20/428). Mean BCVA improved to 1.1 logMAR (SD +/- 0.73 logMAR, Snellen 20/250) at the end of 1 week. One month after surgery the mean BCVA improved to 0.42 logMAR (SD ± 0.35 logMAR, Snellen 20/53), which was statistically significant (*p* = 0.002, Wilcoxon Signed Rank test). The mean BCVA was 0.43 logMAR (SD ± 0.32 logMAR, Snellen 20/54) 3 months after the surgery, which was significantly better than the pre-operative BCVA (*p* = 0.003, Wilcoxon Signed Rank test).

The patients were followed up for a mean period of 7 months (range 1 month to 15 months) after the primary surgery, at which point the BCVA had significantly improved to 0.36 logMAR (SD ± 0.34 logMAR, Snellen 20/46) (*p* = 0.002, Wilcoxon Signed rank test).

Amongst our cases 4 cases (28.5%) were closed under balanced salt solution, 5 cases (35.7%) were closed with an endotamponade of air, 2 cases (14.2%) with octafluoropropane gas (C_3_F_8_) and 3 cases (21.4%) with 1000 centistoke silicone oil.

### Adverse events

Early POVCH occurred in only 1 eye (7.1%) at 1 week post operatively and resolved spontaneously within a month with no intervention such as surgery, anti-VEGF injection or anterior retinal cryopexy. An ERM was noted in 4 eyes (28.5%) after surgery. No instances of delayed POVCH were seen in any case.

None of the patients required any further intervention in the form of laser photocoagulation, intravitreal injections or any ocular surgery during the course of follow up.

Details of the 14 eyes operated is provided in Table 1.

**Table 1:** Details of patients

Serial Number	Age	Systemic co-Morbidities	Pathology	Surgery Performed	Pre-Op BCVA	Post Op BCVA at 1 month	POVCH	Time point of vitreous hemorrhage	Post Op BCVA at last follow up	Period of follow up (Months)
1	47	DM, HTN	PDR with VH	PPV EL Fibrin glue FAE	20/320	20/30	Yes	Post op day 7	20/40	6
2	54	DM, HTN	PDR with VH	PPV EL Fibrin glue FAE	CF-CF	20/30	No		20/25	7
3	47	DM, HTN	PDR with TRD	PPV MP EL Fibrin glue	20/250	20/250	No		20/100	4
4	62	DM, HTN	PDR with VH	PPV EL Fibrin glue FAE	HM	20/50	No		20/100	13
5	64	DM, HTN	PDR with VH	PPV EL Fibrin glue	CF-CF	20/40	No		20/30	11
6	62	HTN	PDR with TRD	PPV MP EL Fibrin glue FAE	20/160	20/200	No		20/200	1
7	37	HTN, TB	Vasculitis with VH	PPV EL Fibrin glue	20/50	20/30	No		20/40	9
8	26		ARN with VH	PPV EL Fibrin glue	20/400	20/20	No		20/20	3
9	63	DM, HTN	PDR with TRD	PPV MP EL Fibrin glue SOI	20/320	20/160	No		20/125	3
10	56	DM, HTN	PDR with TRD	PPV MP EL Fibrin glue SOI	20/160	20/40	No		20/20	12
11	35	HTN	BRVO with VH	PPV EL Fibrin glue FAE	20/400	20/20	No		20/20	5
12	61	DM	PDR with VH	PPV EL Fibrin glue SOI	CF-CF	20/50	No		20/200	5
13	57	DM, HTN	PDR with VH	PPV EL Fibrin glue FGE	20/200	20/50	No		20/50	1
14	63	DM, HTN	PDR with VH	PPV EL Fibrin glue FGE	20/250	20/80	No		20/40	15

Abbreviations: DM: Diabetes mellitus; HTN: hypertension; PDR: Proliferative diabetic retinopathy; ARN: Acute retinal necrosis; BRVO: Branch Retinal Vein Occlusion; VH: Vitreous hemorrhage; TRD: Tractional retinal detachment; PPV: Pars Plana Vitrectomy; EL: Endolaser; FAE: Fluid Air Exchange; MP: Membrane Peeling; SOI: Silicone oil injection; BCVA: Best Corrected Visual Acuity; POVCH: Post-Operative Vitreous Cavity Hemorrhage; CF-CF: Counting Fingers Close to Face; HM: Hand Movements; PL: Perception of Light; PR: Projection of Rays

## Discussion

We have recently published the results of a pilot study, which detailed the benefits if fibrin glue for proliferative diabetic retinopathy [7]. Our current study differs in some key details while also validating the results obtained. It helps prove the reproducibility of the surgical technique [7] as it was conducted at two tertiary care eye centres, with multiple surgeons of different training backgrounds involved. It also gives information about a significantly greater period of follow up (Mean period of follow up of 7 months vs. 3 months). Most importantly the current study focusses only on intractable hemorrhages from the optic disc, whereas the initial pilot study focussed on intractable bleeders in the posterior pole of the retina.

In a retrospective review of patients who underwent PPV for VH due to PDR, POVCH occurred in 37% of the eyes, of which 84% had a recurrent bleeding event within 15 days. It was found that the greater duration of diabetes, lower hemoglobin level, attached status of the posterior vitreous, deficient retinal photocoagulation status, and the presence of TRD were significantly associated with POVCH. In all cases of early rebleed, an active residual fibrovascular membrane was observed [8]. Bleeding from dissected vascular membranes, inadequate intra-operative hemostasis of bleeders and immediate post-operative hypotony are other causes of persistent vitreous hemorrhage in the early post-operative period [3]. 

Persistent bleeding from the optic disc is a very challenging situation. Most vitreoretinal surgeons deal with this potential problem by leaving a stump of neovascularization on the surface of the disc and then apply bipolar diathermy to the fibrovascular tissue to achieve haemostasis.

However a persistent bleed at the disc cannot always be staunched adequately with endodiathermy or with laser photocoagulation, as this could compromise the visual function [9, 10]. Removing adherent membranes from the disc is difficult, whereas inadequate removal of membranes due to the fear of optic bleed is also suboptimal as it leads to recurrent hemorrhages or recurrent tractional retinal detachment [3]. 

Another approach is to raise the infusion pressure beyond 60 mm of Hg. However sometimes bleeding can persist even after multiple attempts of raising infusion pressure.

Fibrin glue is a biological blood-derived tissue adhesive which imitates the final stages of the coagulation cascade. The glue consists of two main components, a solution of concentrated human fibrinogen which is activated by the addition of thrombin and calcium chloride. This results in the formation of a clot within ten seconds to one minute. The clot aids in hemostasis and tissue sealing and gets completely absorbed in 1–2 weeks with minimal fibrosis and without any foreign body reaction [11].

It was shown by Yan et al. that repeat surgery was required in all cases where hemorrhage occurred from the optic disc. This occurred due to residual or recurrent neovascular membrane at the optic disc which could not be removed during the primary surgery [2] Only one of our cases had a recurrent POVCH and even that patient did not need a repeat surgery. We hypothesize that fibrin aids in immediate hemostasis, and in the period during which the fibrin clot has good integrity, the intraoperative pan-retinal laser leads to sufficent fibrosis and quiescence of the neovascular stump still present on the optic nerve head. Thus fibrin glue provides good action against persistent and early POVCH.

In a study by Lee et al. the rate of immediate and recurrent vitreous hemorrhage within the first month was found to be 42.8% and 6.1% respectively [12]. Their criteria were as follows: Immediate POVCH was defined as any hemorrhage that was found from the first postoperative day and persistent POVCH as a subgroup of immediate postoperative POVCH, which was not cleared up until the first postoperative month. Recurrent POVCH was defined as any POVCH occurring after initial clearing of the vitreous cavity within the first 6 postoperative months. As per their criteria the rate of immediate, persistent and recurrent hemorrhage for our cases was 0%, 0% and 7.1% respectively.

The one case to experience POVCH amongst our series was closed with an endotamponade of air. While no statistical association was seen, a higher trend of POVCH was seen in eyes with an endotamponade of gas when assessed by Khuthaila et al [13]. Another study from our institute has shown that there is no difference in the rates of recurrent POVCH, non-resolving POVCH and POVCH requiring repeat surgery. Persistent POVCH in fact may be highest in eyes with the use of silicone oil [14]. The impact of endotamponade on POVCH rates is highly debatable. While this case series is too small to comment upon the relationship between choice of endotamponade and POVCH rates after using fibrin glue, we would like stress that none of the cases closed under balanced salt solution had any rebleeding.

There are concerns regarding the risk of hypersensitivity and viral transmission with the use of fibrin glue [11]. None of the cases in our group’s experience have encountered any adverse effects related to the use of fibrin glue [4–6]. None such issues arose in the current prospective study.

Incidence of ERM in eyes with vitrectomy for diabetic retinopathies has been estimated to vary between 21 to 53% [15, 16]. Rates ranging from 3 to 26% can be seen in the most recent randomized control trials assessing the incidence post-operative ERM formation [17, 18]. In vitro studies have also shown that when a fibrin clot is laid over human retinal pigment epithelial cells, they lose their normal epithelial morphological features and migrate into the overlying clot as fibrocyte like cells [19]. 4 eyes out of the 14 eyes in our series had an incidence of an ERM which was noted at 1 month. However all these eyes had underlying TRD and it is difficult to attribute it to just the use of fibrin glue. None of the eyes in the initial studies where fibrin glue was used for rhegmatogenous retinal detachments (GUARD 1 and GUARD 2) [4, 5] reported the incidence of ERM. Larger studies are therefore needed to establish the incidence of ERM formation after use of fibrin glue in retinal surgeries. It has also been shown that post diabetic vitrectomy recurrence of vitreous hemorrhage may aggravate epiretinal membrane formation, and is a major cause of poor functional recovery post-surgery [15]. By reducing the incidence of POVCH, the use of fibrin glue for persistent optic disc bleeders may in fact reduce the incidence of ERM formation.

One of the major limitations of our study is a lack of comparison or control group to determine if this technique is as good or superior to existing techniques. Other limitations include the heterogeneity in the duration of follow up of the different cases, the different degrees of pre operative pan retinal photocoagulation, the differences in the rates of injection of pre-operative anti-VEGF injection and heterogeneity in the utilisation of any endotamponading agent.

To conclude this case series shows that fibrin glue coagulum provides an adequate sealing effect that prevents persistent ooze from the disc bleed. This technique can be extremely beneficial in the management of intractable bleeders during vitrectomy, providing early as well as long term rehabilitation.

## Supplementary Information

Below is the link to the electronic supplementary material.

Supplementary Material 1

## Data Availability

No datasets were generated or analysed during the current study.

## Declarations

### Presentations

Results presented as a poster at the annual meeting of Association of Research in Vision and Ophthalmology, ARVO 2025 - May 2025 at Salt Lake City, Utah, USA. Surgical Video- Presented at Retina World Congress and was the recipient of the People’s choice award at the Retina World Congress meeting in Fort Lauderdale, Florida, USA in May 2024. Paper on Demand presented in the annual meeting of American society of Retina Specialists (ASRS) in July 2025 at Los Angeles, USA.
