# Correction: Clinical outcomes of fibrin glue assisted hemostasis in persistent intraoperative optic disc bleeding

**DOI:** 10.1186/s40942-026-00827-0

**Published:** 2026-03-09

**Authors:** Saarang Hansraj, Thirumalesh MB, Brijesh Takkar, Vivek Pravin Dave, Ritesh Narula, Srishti Raksheeth Ramamurthy, Mudit Tyagi

**Affiliations:** 1 Smt. Kanuri Santhamma Centre for Vitreoretinal Diseases, Anant Bajaj Retina Institute, Saroja A Rao Uveitis Centre, Kallam Anji Reddy Campus, L V Prasad Eye Institute, Hyderabad, 500034 India; 2 Narayana Nethralaya, Bangalore, India; 3 Indian Health Outcomes, Public Health and Economics Research Centre (IHOPE), LV Prasad Eye Institute, Hyderabad, India

**Correction: International Journal of Retina and Vitreous (2026) 12:33**

10.1186/s40942-026-00801-w

In this article the author’s name Thirumalesh MB was incorrectly written as Thirumalesh.

The original article has been corrected.

